# Jagged Ligands Enhance the Pro-Angiogenic Activity of Multiple Myeloma Cells

**DOI:** 10.3390/cancers12092600

**Published:** 2020-09-11

**Authors:** Maria Teresa Palano, Domenica Giannandrea, Natalia Platonova, Germano Gaudenzi, Monica Falleni, Delfina Tosi, Elena Lesma, Valentina Citro, Michela Colombo, Ilaria Saltarella, Roberto Ria, Nicola Amodio, Elisa Taiana, Antonino Neri, Giovanni Vitale, Raffaella Chiaramonte

**Affiliations:** 1 Department of Health Sciences, Università degli Studi di Milano, 20142 Milano, Italy; mariateresa.palano@multimedica.it (M.T.P.); domenica.giannandrea@unimi.it (D.G.); natalia.platonova@unimi.it (N.P.); monica.falleni@unimi.it (M.F.); delfina.tosi@unimi.it (D.T.); elena.lesma@unimi.it (E.L.); valentina.citro@unimi.it (V.C.); michela.colombo@ndcls.ox.ac.uk (M.C.); 2 Istituto Auxologico Italiano, IRCCS, Laboratory of Geriatric and Oncologic Neuroendocrinology Research, 20095 Cusano Milanino, Italy; germano.gaudenzi@gmail.com (G.G.); giovanni.vitale@unimi.it (G.V.); 3 Department of Biomedical Sciences and Human Oncology, Unit of Internal Medicine and Clinical Oncology, University of Bari Medical School, 70124 Bari, Italy; ilaria.saltarella@libero.it (I.S.); roberto.ria@uniba.it (R.R.); 4 Department of Experimental and Clinical Medicine, Magna Graecia University of Catanzaro, 88100 Catanzaro, Italy; amodio@unicz.it; 5 Department of Oncology and Hemato-Oncology, University of Milano. Hematology, Fondazione Ca’ Granda IRCCS Policlinico, 20122 Milano, Italy; elisa.taiana@unimi.it (E.T.); antonino.neri@unimi.it (A.N.); 6 Department of Clinical Sciences and Community Health (DISCCO), University of Milan, 20122 Milan, Italy

**Keywords:** multiple myeloma, angiogenesis, Notch, Jagged, VEGF, bone marrow stromal cells

## Abstract

**Simple Summary:**

The Jagged family of ligands are aberrantly expressed during multiple myeloma progression and contributes to activate Notch signaling both in myeloma cells and in the nearby bone marrow cell populations activating several pro-tumor effects. This work elucidates, in vitro, in vivo as well as in patients’ bone marrow biopsies, different mechanisms by which tumor cell-derived Jagged1 and 2 contribute to myeloma-associated angiogenesis. These include the ability to induce myeloma and bone marrow stromal cell secretion of VEGF along with a direct activation of the pro-angiogenic Notch signaling pathway in endothelial cells. This research provides a rational for a Jagged-directed therapy in multiple myeloma.

**Abstract:**

Multiple myeloma (MM) is an incurable plasma cell malignancy arising primarily within the bone marrow (BM). During MM progression, different modifications occur in the tumor cells and BM microenvironment, including the angiogenic shift characterized by the increased capability of endothelial cells to organize a network, migrate and express angiogenic factors, including vascular endothelial growth factor (VEGF). Here, we studied the functional outcome of the dysregulation of Notch ligands, Jagged1 and Jagged2, occurring during disease progression, on the angiogenic potential of MM cells and BM stromal cells (BMSCs). Jagged1–2 expression was modulated by RNA interference or soluble peptide administration, and the effects on the MM cell lines’ ability to induce human pulmonary artery cells (HPAECs) angiogenesis or to indirectly increase the BMSC angiogenic potential was analyzed in vitro; in vivo validation was performed on a zebrafish model and MM patients’ BM biopsies. Overall, our results indicate that the MM-derived Jagged ligands (1) increase the tumor cell angiogenic potential by directly triggering Notch activation in the HPAECs or stimulating the release of angiogenic factors, i.e., VEGF; and (2) stimulate the BMSCs to promote angiogenesis through VEGF secretion. The observed pro-angiogenic effect of Notch activation in the BM during MM progression provides further evidence of the potential of a therapy targeting the Jagged ligands.

## 1. Introduction

Multiple myeloma (MM) is a plasma cell malignancy mainly settled in the bone marrow (BM) where it establishes tight communication with the stromal cell populations, including BM stromal cells (BMSCs) and endothelial cells (ECs), and promotes a pro-tumor microenvironment (TME), inducing the angiogenic switch [1] and favoring MM growth and progression [2].

MM may rise as an asymptomatic monoclonal gammopathy of undetermined significance (MGUS), a benign avascular phase of the disease [3]. The progression from MGUS to MM is characterized by clonal expansion of tumor cells within the BM (infiltrating a cell number higher than 10%) and it is coupled with an angiogenic switch that brings forth neo-vessels formation throughout the BM [4]. Consistently, MM-associated endothelial cells (MM-ECs) differ from MGUS-ECs due to their higher intrinsic angiogenic capability [5,6].

Increasing evidence supports the role of the Notch pathway in MM progression [7]. Notch is composed of four trans-membrane receptors (Notch1 to 4) and 2 classes of ligands, named Delta-like (Dll1, 3 and 4) and Jagged (Jagged1 and 2) [8]. The interaction between the ligand and receptor induces two proteolytic cleavages that releases the intracellular portion of Notch, which in turn activates the transcription of the Notch-responsive genes [9].

The Notch pathway plays a relevant role in MM development and progression, mediating the communication between the MM cells and the surrounding cell population of the BM microenvironment, including the BMSCs [10,11] and osteoclasts [12]. This interplay is possibly due to the increased expression of the Jagged ligands on the MM cell surface. Specifically, Jagged2 is already expressed at higher levels in the benign MGUS phase [13], while Jagged1 upregulation occurs later during the progression to the symptomatic MM [14]. Jagged ligands’ increase leads to the aberrant activation of the Notch signaling, not only in MM cells through homotypic interaction, but also in the surrounding BM cells via heterotypic interaction [10,11].

Notch signaling is also involved in physiologic and tumor angiogenesis [15,16,17,18] in coordination with the vascular endothelial growth factor (VEGF)–VEGFR axis [19]. The angiogenic switch in MM, characterized by an increased BM microvessel density (MVD), is associated with a poor prognosis [20] and represents a major event in tumor progression, resulting in increased availability of nutrients and oxygen, necessary for MM cell proliferation, the release of angiocrine factors from the newly formed vessels and a possible way for tumor spread [21].

We recently demonstrated that MM angiogenesis relies on the activation of the Notch pathway in MM-ECs. Notch signaling in MM-ECs is due to the increased expression of the Notch receptor and ligands, resulting in homotypic Notch activation [6]. On the other side, we also observed that MM cell-derived Jagged ligands represents an important source of Notch signaling activation in ECs [6].

In this work, we explored the impact of the aberrant expression of MM-derived Jagged1 and Jagged2 on MM angiogenesis, dissecting their contribution on the angiogenic potential of MM-ECs and BMSCs. Moreover, we investigated the mechanisms mediated by the release of angiogenic factors as well as the direct cell–cell interaction.

## 2. Results

### 2.1. Myeloma Cell-Derived Jagged Ligands Regu Late Myeloma Cell Angiogenic Potential

**Figure 1:**
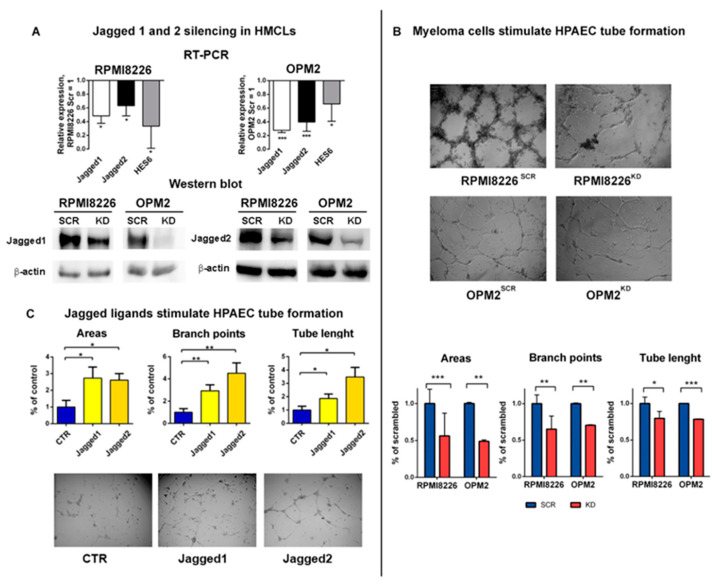
Multiple myeloma (MM) cell-derived Jagged promotes angiogenesis: (**A**) Up: Jagged1 and 2 knock-down (KD) efficiency in RPMI8226^KD^ and OPM2^KD^ cells was obtained by qRT-PCR assay of the relative gene expression variation (normalized to GAPDH) of Jagged1 and Jagged2 and the Notch target gene HES6, calculated by the 2^−ΔΔCt^ formula. Data are expressed as the mean value ± SD. Down: Western blot analysis of Jagged1 and Jagged2 in HMCLs^SCR^ or HCMLs^KD^. Protein loading was normalized to -actin. The shown images are representative of three independent experiments. (**B**) Up: Tube formation assay on Matrigel with co-culture systems of HMCLs^SCR^ or HCMLs^KD^ and primary human pulmonary artery endothelial cells (HPAECs). 4X magnification images are shown. Down: Graphs show quantification of the number of areas and branch points and total tube length. (**C**) HPAEC tube formation stimulated by Jagged1 and Jagged2 peptides. Images are a 4X magnification. Statistical analysis was carried out by a one-tailed t test; * is for *p* ≤ 0.05; ** is for *p* ≤ 0.01; *** is for *p* ≤ 0.001.

To assess the involvement of MM cell-derived Jagged ligands in angiogenesis promotion, we knocked down (KD) the ligands in two human myeloma cell lines (HMCLs), namely RPMI8226 and OPM2, by using siRNAs targeting Jagged1 or Jagged2 (HMCLs^KD^) or the corresponding scrambled siRNA as the control (HMCLs^SCR^), as previously reported [6,10]. We assessed the KD efficacy on Jagged1 and 2 mRNA as well as on the Notch transcriptional targets by using quantitative qRT-PCR (Figure 1A, upper panel) and confirmed the downregulation of the corresponding protein levels by Western blotting (Figure 1A, lower panel).

Our recent findings indicate that the MM cell-derived Jagged1/2 triggered Notch activation in the ECs [6], prompting us to verify if this heterotypic activation could promote angiogenesis. To address this issue, we used different approaches. First, we set up a co-culture system, including HMCLs^SCR^ or HMCLs^KD^ cultured in direct contact with primary human pulmonary artery endothelial cells (HPAECs) laid on a Matrigel-coated support, and explored the changes in the HPAECs’ ability to organize a tube-like network. In Figure 1B, representative images show the tube formation assay (upper panel) and the graphs (lower panel) illustrate the different ability of HMCLs^SCR^ or HMCLs^KD^ to induce HPAECs to form a tube-like network, assessed by counting the number of areas, branch points and the total tube length. HPAECs cultured with both HMCLs^SCR^ displayed a significantly increased complexity of the grid compared to HPAECs cultured with HMCLs^KD^, indicating that the angiogenic potential of the MM cell relies on the expression of Jagged ligands on the MM cells.

This result prompted us to verify if the MM cell-derived Jagged ligands could trigger the angiogenic Notch signaling in HPAECs by direct contact or, instead, if this effect could be mediated by the release of soluble angiogenic factors induced by Jagged-mediated Notch activation through homotypic interaction in the MM cells.

To distinguish between the effect of the MM cell-derived soluble angiogenic factors and the MM cell-derived Jagged-mediated activation of the angiogenic Notch signaling in the HPAECs, we set up a 24 h tube formation assay stimulating the HPAECs with soluble Jagged1 and Jagged2 peptides. The obtained results showed that the Jagged-mediated stimulation might increase the HPAECs’ organizing ability (Figure 1C), and indicated that the MM cell-derived Jagged ligands can engage directly with the Notch receptor on the EC surface and induce its activation as well as the angiogenic response.

**Figure 2:**
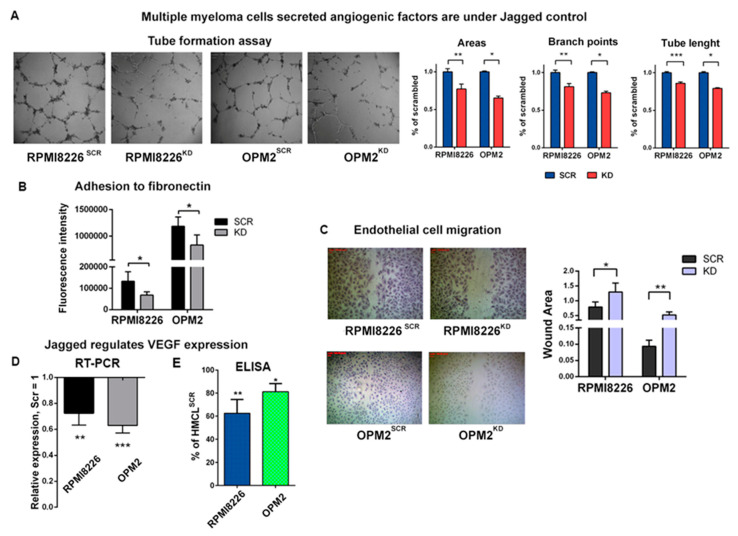
MM cell-derived Jagged promotes angiogenesis: (**A**) Tube formation assay on HPAECs with conditioned media (CM) of HMCLs^SCR^ or HCMLs^KD^. 4X magnification images are shown on the left. Graphs (on the right) show the percentage variation of areas and branch points and total tube length +/-SEM. (**B**) Adhesion to fibronectin of HPAECs treated with CM from HMCLs^SCR^ and HMCLs^KD^ and stained with Calcein-AM. The graph reports the intensity of the adherent fluorescent cells. (**C**) Motility of the HPAECs treated with CM of HMCLs^SCR^ and HMCLs^KD^ was assessed by wound healing assays. Up: Representative pictures at 4X magnification. Down: The graph shows the average open area of the wounds expressed in pixels. (**D**,**E**) Variation of vascular endothelial growth factor (VEGF) expression in HMCLs^SCR^ and HMCLs^KD^ assessed at the mRNA level (**D**) by qRT-PCR of the relative gene expression variation (normalized to GAPDH) calculated by the 2^−ΔΔCt^ formula (data are expressed as the mean value ± SD) and at the protein levels (**E**) by ELISA on 48 h CM. Data are expressed as the amount of VEGF-A released by HMCLs^KD^ normalized on VEGF-A expressed by HMCLs^SCR^. For each sample, the amount of VEGF-A (pg/mL) was normalized to the cell concentration. Statistical analyses were carried out by one-tailed *t*-tests; * is for *p* ≤ 0.05; ** is for *p* ≤ 0.01; *** is for *p* ≤ 0.001.

Since the Jagged ligands can activate Notch signaling also by homotypic interaction in the same MM cells, we wondered if the angiogenic potential of the HMCLs could be ascribed also to a Notch-dependent release of pro-angiogenic soluble factors. To address this issue, we performed a tube formation assay for 24 h on a Matrigel layer with or without the conditioned medium (CM) of HMCLs^SCR^ or HMCLs^KD^. As shown in Figure 2A, the HMCLs^SCR^-derived CM ability to induce HPAECs to organize a grid-like structure is significantly reduced in the presence of HMCLs^KD^-derived CM. We also examined the effect of CM on EC adhesion and migration, two further biological events associated with angiogenesis.

To test EC adhesion, we treated ECs with HMCL-derived CM and assessed the adhesion by measuring the fluorescence intensity after 1 h plating on a fibronectin-coated plate. The results showed a different adhesion capability of the ECs stimulated with CM derived from HMCLs^SCR^ or HMCLs^KD^, since the Jagged ligands’ depletion impaired the EC adhesion, as shown in Figure 2B.

Moreover, the analysis of EC migratory ability confirmed the effects induced by the Jagged KD on the HMCLs. Images and graph in Figure 2C report EC motility after 24 h incubation with the CM derived from HMCLs^SCR^ or HMCLs^KD^. The quantification of the wound area confirmed a reduced motility of the ECs treated with the CM from HMCLs^KD^. Indeed, the wound areas increased by 70% when the ECs were treated with RPMI8226^KD^-derived CM and this effect was even more evident with CM from OPM2^KD^, which showed a 6–7-fold increase in the wound area.

The evidence that Jagged1 and 2 KD reduced the HMCLs’ ability to secrete pro-angiogenic factors in the CM prompted us to assess possible variations in VEGF-A level upon Jagged KD. qRT-PCR analysis showed that the Jagged ligands KD caused a concomitant negative modulation of VEGF-A mRNA in all HMCLs (Figure 2D). An ELISA assay (Figure 2E) confirmed that the down-modulation of VEGF-A mRNA affected also the protein secretion.

Overall, these results indicated that the HMCLs may promote angiogenesis by activating the Notch signaling in ECs via heterotypic Jagged-mediated Notch activation and, additionally, HMCL-mediated secretion of pro-angiogenic VEGF-A is influenced by homotypic activation of the Notch signaling induced by the Jagged ligands. Both these pro-angiogenic ways may be hampered by inhibiting the Jagged-mediated Notch activation.

### 2.2. Myeloma Cell-Derived Jagged Ligands Increase the Angiogenic Potential of Stromal Cells

It is well known that the MM-associated BMSCs play a key role in supporting tumor angiogenesis [22,23] and the interaction between the Notch ligands and receptors is relevant in the communication between MM cells and stromal cells, mediating important effects, such as BMSC-induced drug resistance [10,11]. We wondered if the MM cell-derived Jagged ligands might trigger Notch signaling to boost the pro-angiogenic potential of the BMSCs.

To address this issue, we exploited a co-culture system of the BMSCs and HMCLs^SCR^ or HMCLs^KD^ and analyzed if the ability of tumor cells to activate Notch signaling in stromal cells paralleled a change in the angiogenic potential.

**Figure 3:**
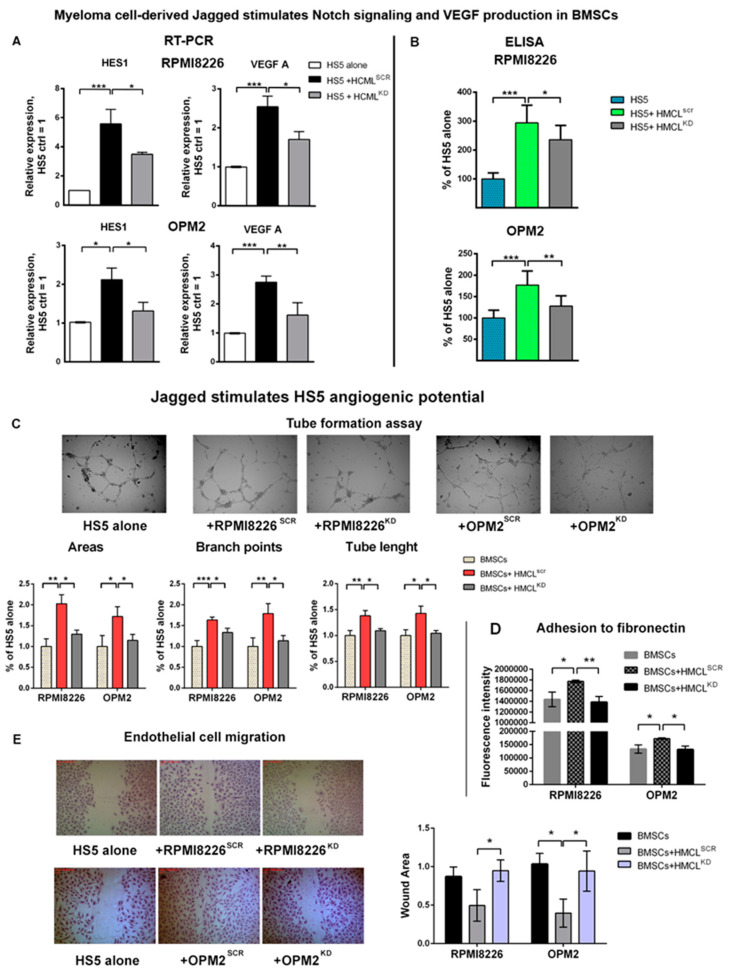
MM cell-derived Jagged stimulates bone marrow stromal cell (BMSC) angiogenic potential: (**A**) Activation of Notch signaling and VEGF-A expression induced in HS5 cells co-cultured with HMCLs^SCR^ or HMCLs^KD^ assessed by qRT-PCR of the relative gene expression variation for the Notch target gene HES1 and VEGF (normalized to HRPT) calculated by the 2^−ΔΔCt^ formula (data are expressed as the mean value ± SD). Statistical analyses were carried out by one-way ANOVA and Tukey post-hoc test; * is for *p* ≤ 0.05; ** is for *p* ≤ 0.01; *** is for *p* ≤ 0.001. (**B**) ELISA for VEGF-A secreted by co-culture systems of HS5 cells and HMCLs^SCR^ or HMCLs^KD^. Data represent the amount of VEGF released by each culture normalized on VEGF expressed by HMCL cultured alone. In each sample, the amount of VEGF (pg/mL) was normalized to the cell concentration. (**C**) Tube formation assay of the HPAECs stimulated with CM secreted by co-culture systems of the HS5 cells and HMCLs^SCR^ or HMCLs^KD^. 4X magnification images are shown. Graphs show the quantification of the number of areas, branch points and total tube length. (**D**) Adhesion to fibronectin of the HPAECs stained with Calcein-AM and treated with CM secreted by the co-culture systems of the HS5 cells and HMCLs^SCR^ or HMCLs^KD^. The graph reports the intensity of the adherent fluorescent cells. (**E**) Migration of the HPAECs treated with CM of co-culture systems of HS5 cells and HMCLs^SCR^ or HMCLs^KD^ was assessed by wound healing assays. Representative pictures at 4X magnification. The graph shows the average open area of the wounds expressed in pixels. Statistical analyses were carried out by ANOVA and Tukey post-hoc tests; * is for *p* ≤ 0.05; ** is for *p* ≤ 0.01; *** is for *p* ≤ 0.001.

First, we assessed if the MM-derived Jagged ligands were able to activate Notch signaling in the BMSCs. To this, we verified if co-cultivating the HMCLs^SCR^ and HMCLs^KD^ with the human BMSC line HS5 induced a variation in the levels of the Notch target genes expression. Results of qRT-PCR analysis in Figure 3A indicate that HMCLs^SCR^ upregulate the transcription of the Notch target gene HES1 in HS5 cells in comparison to the lower basal expression level observed in HS5 cultured alone; on the contrary, the corresponding HMCLs^KD^ displayed a significantly reduced ability to activate HES1 in HS5 cells, consistently with a decreased Notch activation.

In the same cells, we also assessed the possible variation in VEGF-A gene expression and observed that, while HMCLs^SCR^ increased VEGF-A gene expression in HS5 cells, HMCLs^KD^ triggered no or significantly lower levels of VEGF transcription (Figure 3A). Additionally, we showed a consistent decrease in secreted BMSC-derived VEGF-A. At this purpose, VEGF secreted in the CM of HS5 or HS5 cultured with HMCLs^SCR^ or HMCLs^KD^ was assessed by ELISA. Results shown in Figure 3B confirmed that the VEGF-A protein expression in the HS5 cells was upregulated by HMCLs^SCR^, but not or significantly less by HMCLs^KD^, indicating that the MM cells’ ability to induce VEGF-A secretion depends on the MM-cell-derived, Jagged-mediated Notch signaling activation in the BMSCs.

We assessed if the HMCLs’ ability to induce BMSC-mediated VEGF-A through Notch signaling activation had a biological consequence on angiogenesis. For this purpose, we verified if the CM derived from the BMSCs co-cultured with the HMCLs induced a variation in EC ability to form tube-like structures, as well as in their adhesive and migratory properties.

Variations in the BMSCs’ ability to stimulate EC organization was assessed by a tube formation assay after 24 h stimulation with the different CM. Results in Figure 3C show that the CM from the HS5 cells cultured alone displayed an intrinsic ability to stimulate EC organization, but all the parameters are significantly increased by CM from HS5 + HMCLs^SCR^. On the contrary, CM from HS5 + HMCLs^KD^ did not increase the tube organization ability of the HPAECs in comparison to the CM from unstimulated HS5.

To assess the effect on EC adhesion, they were stimulated with the same CM. Results reported in Figure 3D show that the intrinsic stromal cell capability to induce EC adhesion was significantly increased in the presence of CM from HS5+HMCLs^SCR^ but remained unchanged when CM was obtained from HS5+HMCLs^KD^.

Variation in the BMSCs’ ability to induce EC migration after stimulation with HMCLs^SCR^ or HMCLs^KD^ was assessed by performing wound healing assays on HPAECs treated with the CM and measuring the wound open area. Figure 3E shows that motility of the ECs treated with CM from HS5 + HMCLs^SCR^ was higher if compared to CM from HS5 cultured alone or HS5 + HMCLs^KD^. Indeed, CM from HS5+HMCLs^SCR^ decreased the wound area, while the effect of the CM from HS5+HMCLs^KD^ was comparable to that from HS5 cultured alone.

Overall, these results indicated that Notch signaling activation in the BMSCs mediated by the MM-cell-derived Jagged ligands boosted the BMSC angiogenic potential by inducing the release of soluble factors, such as VEGF-A.

### 2.3. Jagged Ligands Promote MM-Associated Angiogenesis in a Zebrafish Model

**Figure 4:**
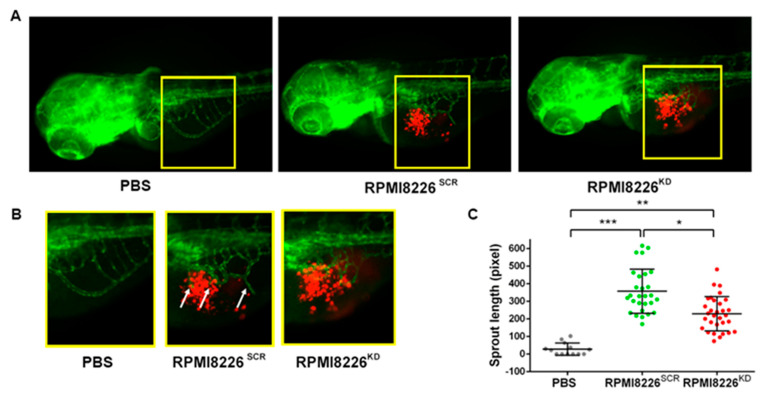
Zebrafish embryo in vivo model to evaluate tumor-induced angiogenesis in relation to Jagged expression in HMCLs. (**A**) Representative images of Tg(fli1a:EGFP)^y1^ zebrafish embryos with GFP expressing vessels (green) and RPMI8226^SCR^ or RPMI8226^KD^ stained with CM-Dil fluorescent dye (red). Epifluorescence images were acquired with a Leica DM 5500B microscope equipped with a DC480 camera. (**B**) Inset of sprouting vessels from SIV in zebrafish embryos 24 hpi; white arrows indicate angiogenic sprouts. (**C**) Quantification of endothelial sprouts from the SIV plexus was performed in 24 h post-injection (hpi) zebrafish embryos injected with RPMI8226^SCR^ (N = 24) and RPMI8226^KD^ cells (N = 31) using ImageJ software (National Institutes of Health, USA). Statistical analysis was carried out by one-way ANOVA with Tukey post hoc tests of three independent experiments; * is for *p* ≤ 0.05; ** is for *p* ≤ 0.01; *** is for *p* ≤ 0.001.

To further investigate the role of the Notch pathway in angiogenesis stimulation and to confirm our in vitro results, we investigated the effect of Jagged1 and Jagged2 in an in vivo zebrafish embryonic model. The transgenic zebrafish model *Tg (fli1a:EGFP)^y1^* allows the detection of ECs in blood vessels with constitutive EGFP expression. RPMI8226^SCR^ or RPMI8226^KD^ cells, pre-stained with the red fluorescent cell tracker CM-Dil, were grafted in 48 h post fertilization (hpf) zebrafish embryos. As negative control of the implantation, embryos were injected with PBS, the cell resuspension medium. Injection was performed in the sub-peridermal space, close to the sub-intestinal vein (SIV) plexus. The sprouting of tumor-induced endothelial structures from the SIV toward the tumor engraftment was evaluated 24 h post-injection (hpi). Injected RPMI8226 cells displayed a comparable localization at 24 hpi (Figure 4A) and length measurements of the vessels arising from SIV (Figure 4B) indicated different stimulation capabilities. Fish embryos receiving RPMI8226^SCR^ cells displayed a significant increase in angiogenic sprouts, which were not visible in embryos receiving PBS, confirming the MM cells’ ability to stimulate angiogenesis. On the contrary, RPMI8226^KD^ cells displayed a mild stimulation of angiogenesis, approximately 35% less RPMI8226^SCR^ cells, indicating that the decrease in Jagged expression in the MM cells impairs their ability to stimulate sprout angiogenesis.

### 2.4. Identification of a Correlation between Jagged Expression in MM Cells and Angiogenesis in Patients’ Bone Marrow

To validate in vitro and in vivo findings, BM biopsies (BMBs) from 34 MM patients were evaluated. Patients analyzed were at the onset of the disease and had not received any drug treatment. Clinical information concerning tumor BM infiltration by MM cells was associated with Jagged1 and Jagged2 expression, Notch activation and angiogenesis investigated by immunohistochemistry.

**Figure 5:**
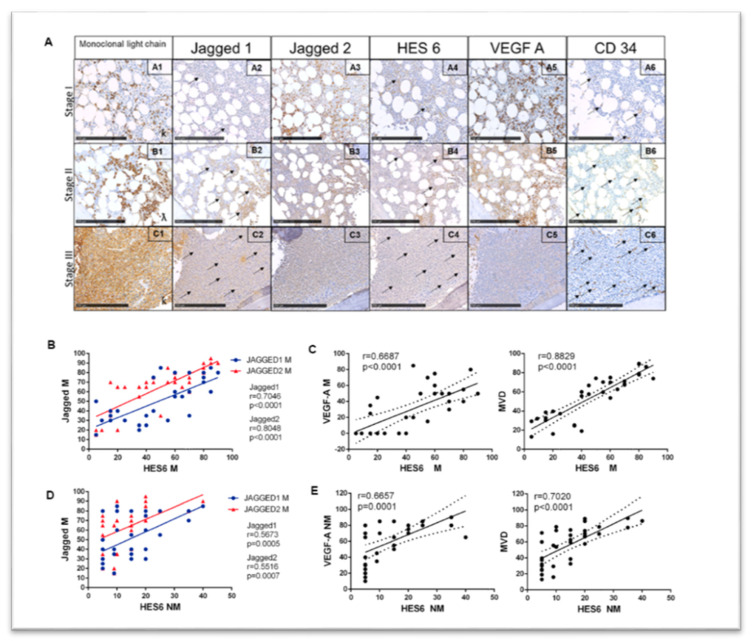
Correlation analysis of the Jagged ligands, Notch activation and angiogenesis in MM patients’ BMBs. (**A**) representative images of the antigen immunoreactivity for the monoclonal Ig light chain, Jagged1, Jagged2, HES6, VEGF-A and CD34 in BMBs from 34 MM patients at different degrees of tumor infiltration (I: less than 20%; II: 21–50%; III: >51%). The arrows indicate specific immunoreactive signals. Photos were acquired at Nano-Zoomer 2.0 and scale bar is for 250 µm. Pearson’s correlation coefficient (r) and the *p*-values are reported for the correlation analyses between (**B**) Jagged1 and Jagged 2 expressed in MM cells and the Notch transcriptional target HES6 in the same tumor cells; (**C**) HES6 and VEGF-A expressed in MM cells or MVD; (**D**) Jagged1 and Jagged 2 expressed in MM cells and the Notch transcriptional target HES6 in nearby non-tumor cells; and (**E**) HES6 in non-tumor cells and VEGF-A in non-tumor cells and MVD. M = MM cells; NM = non tumor cells. Statistical analysis was carried out using two-tailed *t*-tests.

Antigen immunoreactivity was evaluated both in the MM cells and non-tumor cell populations. Results are tabulated in Table 1 and shown in Figure 5.

As expected, in neoplastic areas, new vessels were very small in the early stages of MM infiltration and progressively increased in number and size, showing bigger lumina and strong CD34 immunoreactivity in plump endothelium with higher levels of infiltration (Figure 5A). All the markers analyzed increased with tumor infiltration levels in BMBs, with some differences (Figure 5A and Table 1): Jagged2 was expressed with higher intensity in MM cells at a low level of tumor infiltration in comparison with Jagged1, whose expression was weaker in infiltration degree I and showed a more evident progressive increase at higher infiltration levels. Similarly, VEGF-A expression in the MM cells showed an increasing trend.

To confirm that the Jagged ligand-directed Notch activation in MM cells was associated with an increased angiogenic potential, we performed the correlation analyses reported in Figure 5B,C and Supplementary Table S1. Findings indicate that in the MM cells the expression of both Jagged1 and Jagged2 correlates with HES6 immunoreactivity, an index of Notch pathway activation [7], with r = 0.7046 and r = 0.8048, respectively. In turn, the HES6 levels in the MM cells significantly correlates with VEGF-A expression and MVD. Jagged ligands immunoreactivity also directly correlated with MM cell-derived VEGF-A and MVD (Table S1).

We also validated the Jagged-mediated MM cells’ ability to boost the BMSC angiogenic potential by examining the correlation of MM-derived Jagged ligands with the immunoreactivity of the other markers on stromal non-tumor cells. As reported in Figure 5D–E, Jagged1 and Jagged2 expressed in the MM cells correlated with the HES6 expression in non-tumor cells (r = 0.5673 and r = 0.5516, respectively), confirming a role of MM cell-derived Jagged in activating the Notch signaling in stromal cells. In turn, HES6 immunoreactivity was associated with VEGF-A expression in non-tumor cells and MVD, suggesting that Notch activation in stromal cells contributes to induce angiogenesis.

On the whole, the immunochemistry results of the MM patients’ BMBs appear to be consistent with in vitro and in vivo evidence.

## 3. Discussion

The interplay between malignant plasma cells and the BM cell populations has been extensively studied in the past years since it is a key step in MM progression and an important target for new anti-tumor therapeutic approaches [24].

Notch signaling plays a pleiotropic role in different cellular contexts; it mediates cell–cell communication in cell fate decision, stem cell maintenance, cell proliferation and survival [25]. Mutations or aberrant expression of the Notch pathway members lead to increased signaling and are frequently present in solid and hematologic cancers [8]. In MM, the widespread Notch signaling activation characterizes the whole TME with important consequences ranging from drug resistance [10,26,27,28] to proliferation [7,29] and bone disease [12,30].

MM cell-derived Jagged ligands play a key role in the activation of Notch signaling in the BM cell populations. Accordingly, Jagged1 expression significantly correlates with disease progression, as we and others previously reported [7,14], while Jagged2 deregulation is an early event occurring at the benign MGUS phase [13], which results from both changes in the transcription level [13,31] or aberrant expression of Skeletrophin, an ubiquitin ligase that regulates Jagged2 activity [32].

Given the relevance of Jagged ligands in this disease, here we have analyzed the role of the Notch pathway in MM-induced angiogenesis, a key feature of MM progression that involves the communication with BM resident cell populations.

The angiogenic switch represents an important change in the BM of MM patients and fosters tumor growth and dissemination. The BM circulation is maintained by different stimuli provided by BM resident cells [33]. It is now well established that the accumulation of malignant plasma cells in the BM destabilizes this balance, leading to neovascularization, which in turn contributes to tumor progression [33].

We recently showed that Notch signaling in the ECs is necessary for angiogenesis activation since the inhibition of Notch1 and 2 activity in the ECs hampered their angiogenic potential [6]. Notch activation in the ECs may be triggered by homotypic activation due to the increased expression of Notch receptors and ligands in MM-ECs during the progression from MGUS to MM [6]. Additionally, we showed that the hyperexpression of Jagged1 and 2 ligands, which can be observed during MM cell progression [7,13,14], may trigger Notch activation in ECs by heterotypic interaction [6].

The importance of Jagged1 and 2 dysregulated expression in MM prompted us to investigate their role in MM angiogenesis exploring their effect on Notch signaling activation in ECs as well as their ability to stimulate the angiogenic potential of the BMSCs.

To study the direct effect of MM-derived Jagged on tumor angiogenesis, we interfered with the Jagged1 and Jagged2 expression in two MM cell lines, RPMI8226 and OPM2, and demonstrated that their silencing was associated with a reduced ability of MM cells to induce HPAEC organization in tube-like structures. The angiogenic stimulus provided by the MM cells was dual: it could be mediated by a direct cell-to-cell contact or by soluble tumor-derived factors. Indeed, we demonstrated that the administration of Jagged1 and 2 peptides triggered the activation of Notch signaling in ECs stimulating angiogenesis. This indicated that the Jagged ligands on the surface of the MM cells were able to activate the angiogenic signals in nearby ECs. On the other side, Jagged-mediated Notch signaling activation in MM cells could promote the secretion of soluble factors, activating the EC angiogenic potential. In fact, the CM from MM cells increased the ECs’ ability to form tube-like structures, promoting their adhesion and migration. The observed effect relied on Jagged-induced Notch activity in MM cells, since the CM from HMCLs^KD^ displayed a significantly reduced angiogenic potential, indicating that the release of MM-derived angiogenic factors depends on Notch signaling activation. VEGF-A was identified as a Notch-dependent angiogenic soluble factor released from MM cells upon Jagged-mediated Notch signaling activation, since its mRNA and protein level were inhibited by Jagged1 and 2 KD.

Additionally, since tumor-angiogenesis is a team effort of BM resident cells characterizing the TME, we also investigated the contribution of BMSCs, which display important angiogenic properties. We verified if the BMSC angiogenic properties could be increased by MM-cell-derived Jagged ligands by using a co-culture system, including HMCLs and the human BM stromal cell line, HS5. We found that the BMSCs pre-stimulated with HMCLs increased their ability to release soluble factors, which could activate the angiogenic potential of the HPAECs, assessed as tube organization ability, motility and adhesion to fibronectin.

We showed that this effect was dependent on the MM-cell-derived Jagged ligands’ ability to trigger Notch signaling in the BMSCs. As a matter of fact, when the HMCLs were depleted of Jagged1 and 2, the ability to trigger Notch signaling in the BMSCs and to stimulate their pro-angiogenic activity was significantly reduced. Additionally, since the BMSCs represent the major source of VEGF-A in the BM, we verified if the Notch signaling, activated by the MM-cell-derived Jagged ligands, was associated with an increase in VEGF-A secretion by the BMSCs. Our results confirmed that the control HMCLs^SCR^ were able to induce VEGF-A secretion by the BMSCs, while HMCL^KD^ did not.

The obtained results clearly show that the MM-derived Jagged ligands may unbalance the levels in Notch activation in the different cell populations, including the MM cells, ECs and BMSCs, thus spreading widely the angiogenic potential in the TME.

To validate in vivo the role played by Notch signaling in MM-associated angiogenesis, we used the zebrafish embryo model *Tg(fli1a:EGFP)^y^^1^*, which allows to detect the formation of new vessels sprouting from the sub-intestinal plexus 24 h following the injection of HMCLs^SCR^ or HMCL^KD^. The evidence that the angiogenic sprout induced by control HMCLs^SCR^ was significantly decreased upon Jagged1 and 2 KD prompted us to hypothesize that also in vivo MM-cell-directed angiogenesis relies on the expression of Jagged1 and 2 in MM cells.

Finally, by analyzing the MM patients’ BMBs, we confirmed that the Jagged ligands expressed by the tumor cells are associated with the diffusion of Notch activation in the whole TME, including tumor and non-tumor cells. We found a strong positive correlation among the Jagged ligand levels in MM cells and Notch signaling activation (HES6 expression) in MM cells, as well as in nearby non-tumor stromal cells. Moreover, Notch activation significantly correlated with the expression of VEGF-A in the same cells, and also with MVD. These results show that such an association occurs in MM patient’s BM between the activation of Notch in tumor cells, TME and the angiogenic switch.

The result is even more interesting considering the significant correlation between the level of MM cells BM infiltration, the expression of Jagged ligands in MM cells and the increase in MVD, which associate the aberrant expression of Jagged1 and 2 with the angiogenic switch occurring during MM progression. Indeed, our evidence indicates that the Jagged ligands’ levels increase with BMB infiltration; in particular, while Jagged1 expression rises slowly and progressively increases, Jagged2 is already expressed at higher levels in the BMBs with the lower levels of infiltration (Table 1). This is consistent with other pieces of evidence, including (1) our recent analysis of tumor cells from MM patients’ gene expression profiling, which indicated that Jagged1 and the Notch transcriptional target HES5 are overexpressed in tumor cells compared to healthy controls, and reach a higher expression level in the more aggressive primary plasma cell leukemia [7]; (2) a refined immunohistochemical analysis revealed that Jagged1 dysregulation occurs during the progression from monoclonal gammopathy of uncertain significance (MGUS) to MM [14]; and (3) Jagged2 overexpression is an earlier event that precedes MGUS and correlates with disease progression [13].

The obtained results contribute to provide a more comprehensive picture of the major drivers of the angiogenic switch during MM progression and of the role played by the Notch pathway: MM cells increase the expression levels of Jagged1, 2 and Notch1 and 2 receptors, promoting (1) homotypic activation of Notch signaling in MM cells that, in turn, results in the secretion of VEGF; (2) heterotypic activation of the angiogenic Notch signaling in ECs; and (3) heterotypic activation of Notch signaling in BMSCs that, in turn, boosts the secretion of VEGF, inducing a potent angiogenic signal. It should be noted that BMSCs may release a higher amount of VEGF in comparison with MM cells (our ELISA results indicate that they may release 2035 ± 96 pg/mL of VEGF compared to 938 ± 72 pg/mL for RPMI8226 and 797 ± 85 pg/mL for OPM2 cells, even if in our experimental conditions the HCMLs were maintained four times more concentrated than the HS5 cell line. Additionally, we demonstrated that the BMSC-mediated VEGF secretion may be further increased by MM cell stimulation. Therefore, we believe that the more effective angiogenic effect of the Notch signaling widespread in the TME is probably mediated by MM-stimulated BMSCs. This effect may be further enhanced by the previously reported increase in Notch receptors and ligands expression occurring in the ECs during MM progression [6]. This may intensify the homotypic interactions between the ECs and provide a greater availability of Notch1 and 2 receptors for the interaction with MM-cell-derived Jagged1 and 2.

In conclusion, the tight spatial and temporal regulation of Notch ligands necessary for the balanced differentiation of new vessels seems to be deranged by the accumulation of MM cells and MM-derived Jagged ligands.

## 4. Materials and Methods

### 4.1. Cell Lines

The HMCLs used in this study, RPMI8226 (ATCC^®^ CCL-155TM) and OPM2 (ACC-50), were cultured in RPMI1640 medium (Lonza, Swiss) supplemented with 10% fetal bovine serum (FBS) (Euroclone S.p.A., Italy), 100 U/mL P/S (penicillin/streptomycin) (Microgem, IT, USA) and 2 mM L-glutamine (Microgem, Italy). Cell lines were seeded at 3 × 10^5^ cells/mL and split every 48 h. HPAECs (Human Pulmonary Artery endothelial cells—ATCC^®^ PCS-100-022TM) were cultured in Vascular Basal medium (ATCC^®^ PCS-100-030TM) supplemented with the Endothelial Cells Growth Kit-VEGF (ATCC^®^ PCS-100-041TM), following the manufacturer’s instruction (complete vascular medium). The HPAECs were seeded at a final concentration of 3 × 10^3^ cells/cm^2^. The human BMSC line HS5 (ATCC^®^ CRL-11882™) was cultured in DMEM (Lonza, Swiss) supplemented with 10% FBS, 100 U/mL P/S and 2 mM L-glutamine.

### 4.2. HMCL Knockdown and Co-Culture Experiments

HMCLs were transfected using specific siRNAs directed against Jagged1 and Jagged2 at a final concentration of 25 nM each and a Lipofectamine RNAiMAX Transfection Reagent [10]. For the co-culture system of the HS5 cell line with transfected HMCLs, HS5 cells were seeded in 24-well plates at a density of 1.5 × 10^5^ cells/mL, and transfected HMCLs were added at the concentration 6 × 10^5^ cells/mL (ratio 1:4). Cells were co-cultured for 40 h and the CM of the last 24 h was analyzed by ELISA or used for tube formation assay. HS5 cells were collected and extracted RNA was analyzed by qRT-PCR. For the in vivo experiments, the HEK293 cell line was transfected using CaCl_2_ with the pTRIPZ vector carrying a doxycycline-inducible system expressing shRNA against Jagged1 and Jagged2 or the corresponding scramble shRNA (Thermofisher, MA, USA). RPMI8226 infection with lentiviral particles were carried out in the presence of 10 μg/mL polybrene (Sigma Aldrich), 20 ng/mL IL6 (Peprotech Inc., USA) and 20 ng/mL IGF1 (Peprotech Inc., USA). After 48 h, the infected cells were selected with puromycin 1 µg/mL. Before injection, the cells were stimulated with doxycycline, 1 µg/mL for 72 h.

### 4.3. Tube Formation Assay

Reduced Matrigel (Corning, NY, USA) was dispensed in a 96-well plate, 50 µL/well, and incubated for 1 h at 37 °C. The HPAECs were seeded on Matrigel-coated wells at 2 × 10^4^ cells/well. The assay was carried out using different stimuli as follows: (i) HMCLs^SCR^ or HMCLs^KD^ were seedead on a layer of HPAECs at a ratio 1:2 (HPAECs:HMCLs); (ii) 100 uL CM from HMCLs^SCR^ and HMCLs^KD^ or CM from co-cultures systems of HS5 cells and HMCLs^SCR^ or HMCLs^KD^ were dispensed in each well; (iii) human recombinant Jagged1 (#188-204—AnaSpec) or Jagged2 (R&D Systems Inc., Minneapolis, MN, USA) were added to RPMI1640 medium at the concentration of 20 µg/mL according to manufacturer’s instruction. Photos of the tube-like structures were acquired after overnight incubation with the EVOS-inverted microscope (Euroclone S.p.A., Italy) or PrimoVert Microscope (Zeiss, Germany) at ×4 magnification. Numbers of area, numbers of branch points and total tube length were analyzed using the ImageJ program.

### 4.4. Adhesion Assay

HPAECs were seeded in a 6-well plate at 3 × 10^3^ cells/cm^2^ density. After 24 h, the medium was changed with CM from HMCLs^SCR^ and HMCLs^KD^ cultured alone or co-cultured with the HS5 cell line for 72 h and diluted 1:1 with fresh RPMI1640 medium supplemented with 2% FBS, 100 U/mL P/S and 2 mM L-glutamine. The HPAECs were incubated for 24 h and a black 96-well plate was coated over night at 37 °C with 100 µg/mL fibronectin diluted in 0.005M Tris-HCl, pH 7.4. The day after the HPAECs were stained with 5 µM Calcein AM 1 h at 37 °C and were seeded on fibronectin at 2.5 × 10^4^ cells/well in fresh-RPMI1640 for 1 h in an incubator at 37 °C with 5% CO_2_. The fluorescence intensity was read with an Ensight Multimode Plate Reader (Perkin Elmer, Inc. MA, USA).

### 4.5. Wound Healing Assay

The HPAECs were seeded in a 48-well plate in complete vascular medium in order to have a confluent well after 48 h. When cells reached confluence, the medium was substituted with CM from HMCLs^SCR^ and HMCLs^KD^ cultured alone or co-cultured with the HS5 cell line for 72 h and diluted 1:1 with fresh RPMI1640 medium supplemented with 2% FBS, 100 U/mL P/S, 2mM L-glutamine and the wound was done using a p200 tip. After 24 h incubation, the HPAECs were washed once with 1× PBS, stained with Comassie Blue and the photos were acquired with a PrimoVert Microscope (Zeiss, Germany) at ×4 magnification. Migration was determined using the ImageJ program as an average open area of the wound.

### 4.6. RNA Extraction and qRT-PCR

Total RNA samples from the HS5 cells cultured alone or with HMCLs^SCR^ or HMCLs^KD^ were extracted by TRI Reagent (Merck, Italy), following the manufacturer’s instructions. Total RNA (1 µg) was retrotranscribed using RevertAid M-MuLV Reverse Transcriptase (ThermoFisher). Quantitative PCR (qRT-PCR) reactions were carried out on a Step-One Plus PCR system (Applied Biosystems, Life Technologies Italia, Italy) using GoTaq^®^ qPCR (Promega, Italy). The primer sequences are reported in Table 2.

### 4.7. ELISA for VEGF-A

VEGF-A ELISA kit (Thermo Scientific) was used to detect human VEGF-A in supernatants of single cultures or co-cultures diluted 10 times, following manufacturer instructions. All analyses were performed in duplicate. Secreted VEGF protein concentration was normalized to the numbers of cells. For co-cultures, the total VEGF protein content was subtracted from the VEGF protein content of the HMCLs and normalized to the number of HS5 cells.

### 4.8. Zebrafish Injection

Adult zebrafish (*Danio rerio*) were maintained according to European laws (2010/63/EU and 86/609/EEC). The 48-hpf *Tg(fli1a:EGFP)^y1^* transgenic embryos [34] were implanted with 72 h-induced HMCLs^SCR^ or HMCLs^J1/2KD^ cells, using a previously described procedure for neuroendocrine tumors [35]. In brief, cells were labeled with a red fluorescent viable dye (CM-Dil, Molecular Probes-Invitrogen, MA, USA) and resuspended with PBS. The 48 hpf zebrafish embryos were anesthetized with 0.02 mg/mL tricaine and injected with stained tumor cells (about 600 cells in each embryo) into the sub-peridermal space, close to the developing SIV plexus. At 24 h hpi, epifluorescence images were acquired with a Leica DM 5500B microscope equipped with a DC480 camera. The cumulative length of the tumor-induced endothelial structures, sprouting from the SIV plexus, were measured in each implanted embryo using ImageJ software. 

### 4.9. Immunohistochemical Analysis of Human Bone Marrow Biopsies

Archival BMBs from 34 MM patients diagnosed at the Unit of Pathology, A.O. San Paolo, Department of Health Sciences, University of Milan, Italy were evaluated in the present study approved by the Ethical Committee of Milano University (No. 8/15—4 March 2015).

Histopathological diagnosis of MM was carried out according to the WHO classification criteria; BMPs were subdivided according to the extent of the BM infiltration by the myeloma cells as follows: degree of infiltration I = less than 20% (10 cases); II = 21–50% (11 cases), III > 51% (13 cases). Notch activation and angiogenesis were investigated by immunohistochemistry (IHC) with an automatic immunostainer (DAKO OMNIS) using diaminobenzidine as the chromogen. Used antibodies were reported in Table 3. Digital images were obtained by the NanoZoomer 2.0 scanner (Hamamatsu Photonics, Japan).

Statistical analyses were performed using GraphPad Prism 6 software (GraphPad software, San Diego, CA, USA). For the in vitro assays including two groups, we carried out one-tailed Student’s *t*-tests; when including 3 or more groups, we performed a one-way ANOVA with Tukey’s post-hoc tests. For in vivo experiments, the minimum size of each group was determined using an a priori power analysis for a one-way ANOVA with an alpha = 0.05 with G-power 3.2 software.

## 5. Conclusions

Jagged ligands dysregulation is frequent in different types of tumors and is often reported to influence angiogenesis in different neoplasms besides MM, i.e., breast cancer [36,37,38] and head and neck squamous cell carcinoma [39]. Boareto and colleagues reported that Jagged ligand expression mediates the differences between physiologic and tumor angiogenesis by affecting the tip-stalk cell fate decisions during vessel formation. Indeed, normal angiogenesis is characterized by a balanced expression of Notch ligands where Dll ligands, expressed in the tip cells, drive new vessels sprouting, and Jagged1 and 2 in the stalk cells drive vessel elongation. In contrast, Jagged overexpression observed in MM and in other types of tumors results in a hybrid tip/stalk phenotype characterized by compromised migration traits compared to tip cells and therefore leading to smaller and poorly perfused vessels compared to those led by the tip cells [40].

Overall, the Jagged ligands’ overexpression has a pleiotropic effect, influencing tumor biology and the pathological communication with the TME. This also includes the promotion of a key step in tumor progression, such as MM-associated angiogenesis, and further strengthen the indication for a therapeutic approach directed to inhibit Jagged-mediated Notch activation by specific monoclonal antibodies [41] or small molecules [42].

## Figures and Tables

**Table 1:** Immunohistochemical analysis of the Jagged ligands, Notch activation and angiogenesis in MM patients’ bone marrow biopsies (BMBs). The degree of malignant plasma cell infiltration in the BM is reported along with the antigen immunoreactivity for monoclonal or Ig light chains. Additionally, the percentage of immunoreactive cells out of the total cells for Jagged1 and 2, HES6 and VEGF-A is reported both for MM (M) and non-MM (NM) cells, along with the microvessel density (MVD) evaluated as the average number of cell determinant 34+ (CD34+) vessels per field.

PATIENT N.	INFILTRATION DEGREE	LIGHT CHAIN	JAGGED1 M	JAGGED1 NM	JAGGED2 M	JAGGED2 NM	HES6 M	HES6 NM	VEGF-A M	VEGF-A NM	MVD
1	I	K	50	5	20	15	5	5	0	30	13.0
2	I	L	30	5	35	40	15	5	0	40	30.3
3	I	L	30	9	20	25	9	5	0	15	32.0
4	I	L	40	9	20	20	20	9	0	35	16.0
5	I	L	15	9	15	20	5	9	0	35	29.3
6	I	L	20	5	65	35	35	5	0	31	24.7
7	I	K	25	5	70	40	40	5	0	10	19.0
8	I	K	35	9	65	80	40	9	0	45	55.7
9	I	L	30	9	65	60	25	5	0	20	37.3
10	I	L	25	9	55	30	35	5	0	25	25.3
11	II	L	25	15	65	40	40	5	20	70	60.0
12	II	K	30	15	70	45	55	15	15	50	59.7
13	II	L	35	20	70	70	15	15	35	65	38.7
14	II	K	40	35	65	75	20	5	45	80	39.7
15	II	K	40	25	70	50	45	20	20	75	57.7
16	II	K	40	25	40	55	15	15	25	55	32.7
17	II	K	85	15	35	80	50	10	50	70	74.3
18	II	K	60	40	70	75	60	10	60	70	53.7
19	II	K	80	30	75	55	70	5	30	65	71.3
20	II	L	55	25	65	80	65	15	50	65	62.7
21	II	K	60	50	70	85	70	20	40	70	67.3
22	III	K	30	25	85	85	55	20	70	80	70.3
23	III	K	80	55	75	90	60	15	75	85	75.0
24	III	L	65	25	60	80	70	15	50	85	68.3
25	III	L	80	30	90	85	80	10	55	85	79.0
26	III	K	70	30	95	90	85	20	80	80	86.0
27	III	K	75	25	75	80	45	20	85	70	66.7
28	III	K	55	30	80	80	60	25	50	85	70.0
29	III	K	80	50	80	90	80	35	40	90	78.0
30	III	K	80	30	80	95	N.A.	25	45	80	79.0
31	III	K	75	25	85	95	80	20	40	80	89.0
32	III	L	85	55	90	90	85	40	80	65	86.3
33	III	K	70	45	80	85	80	35	40	80	89.7
34	III	K	80	30	90	85	90	20	50	70	74.0

**Table 2:** Sequences of the primers used for the qRT-PCR.

hGAPDH	5′-ACAGTCAGCCG ATC TTC TT-3′	5′-AATGGAGGGGTCATTGATGG-3′
h18S	5′-GTAACCCGTTGAACCCCATT-3′	5′-CCATCCAATCGGTAGTAGCG-3′
hJagged1	5′-GCAACACCTTCAACCTCAAG-3′	5′-GTTGAACGGTGTCATTACTGG-3′
hJagged2	5′-TCATCCCCTTCCAGTTCG-3′	5′-TGGTATCGTTGTCCCAGTC-3′
hHES1	5′-AGGCGGACATTCTGGAAATG-3′	5′-CGGTACTTCCCCAGCACACTT-3′
hHES6	5′-CGTGAGGATGAGGACGG-3′	5′-AGGCTCTCGTTGATCCG-3′
hVEGF-A	5′-GGGCAGAATCATCACGAAGT-3′	5′-TGGTGATGTTGGACTCCTCA-3′
hHPRT	5′-TTTATGTCCCCTGTTGACTGGT-3′	5′-GTAGCCCTCTGTGTGCTCAA-3′

**Table 3:** Antibodies and experimental conditions used for the IHC.

Antigen	Clone	Source	Dilution/Time	Unmasking
Kappa light chain	-	Agilent	1:10; 1 h	FLEX TRS Low pH
Lambda light chain	-	Agilent	1:10; 1 h	FLEX TRS Low pH
Jagged 1	AF1277 Goat	R&D syst.	1:100; 1 h	EDTA
Jagged 2	4F10 Mouse	Santa Cruz	1:200; o.n.	Citrate
HES6	Polyclonal Rabbit	Abcam	1:300; o.n.	Citrate
VEGF-A	A-20 Polyclonal Rabbit	Santa Cruz	1:800; 1 h	Citrate
CD34	QBEnd 10 Monoclonal Mouse	Agilent	Ready-to-Use	FLEX TRS Low pH

Statistical analysis and percentage variation among the HMCL experimental conditions.

## Supplementary Materials

The following are available online at https://www.mdpi.com/2072-6694/12/9/2600/s1, Table S1: Correlation matrix of markers for ligand and target gene of Notch pathway and angiogenesis quantified in MM patients’ BM biopsies.
