# Supplementary material for: Jagged Ligands Enhance the Pro-Angiogenic Activity of Multiple Myeloma Cells
Source: Cancers (Basel). 2020 Sep 11;12(9):2600. doi: 10.3390/cancers12092600 (PMC7565520; doi:10.3390/cancers12092600)
Supplement: Supplementary file 1 [file cancers-12-02600-s001.pdf]

Supplementary Materials: Jagged Ligands Enhance the Pro-Angiogenic Activity of Multiple Myeloma Cells

Maria Teresa Palano, Domenica Giannandrea, Natalia Platonova, Germano Gaudenzi, Monica Falleni, Delfina Tosi, Elena Lesma, Valentina Citro, Michela Colombo, Ilaria Saltarella, Roberto Ria, Nicola Amodio, Elisa Taiana, Antonino Neri, Giovanni Vitale and Raffaella Chiaramonte

Table 1. Correlation matrix of markers for ligand and target gene of Notch pathway and angiogenesis quantified in MM patients' BM biopsies.

**Pearson Correlation Analysis**		**MM Stage**	**JAGGE D1 NM**	**JAGGE D1 M**	**JAGGE D2 NM**	**JAGGED2 M**	**HES6 N M**	**HES6 M**	**VEGF-ANM**	**VEGF-A M**	**MVD**
MM stage	coefficient r		0.7533	0.6970	0.8280	0.7316	0.7461	0.7839	0.8920	0.8734	0,8787
	p-value		<0.0001	<0.0001	<0.0001	<0.0001	<0.0001	<0.0001	<0.0001	<0.0001	<0.0001
JAGGED1 NM	coefficient r	0.7533		0.6972	0.7360	0.6012	0.7223	0.6822	0.7511	0.7519	0,7169
	p-value	<0.0001		<0.0001	<0.0001	0.0002	<0.0001	<0.0001	<0.0001	<0.0001	<0.0001
JAGGED1 M	coefficient r	0.6970	0.6972		0.6926	0.4797	0.5673	0.7046	0.6287	0.6845	0,7424
	p-value	<0.0001	<0.0001		<0.0001	0.0041	0.0005	<0.0001	0.0001	<0.0001	<0.0001
JAGGED2 NM	coefficient r	0.8280	0.7360	0.6926		0.7677	0.6498	0.7661	0.8012	0.8117	0,8564
	p-value	<0.0001	<0.0001	<0.0001		<0.0001	<0.0001	<0.0001	<0.0001	<0.0001	<0.0001
JAGGED2 M	coefficient r	0.7316	0.6012	0.4797	0.7677		0.5516	0.8048	0.6233	0.6443	0,7368
	p-value	<0.0001	0.0002	0.0041	<0.0001		0.0007	<0.0001	0.0001	<0.0001	<0.0001
HES6NM	coefficient r	0.7461	0.7223	0.5673	0.6498	0.5516		0.6439	0.6057	0.5956	0,7020
	p-value	<0.0001	<0.0001	0.0005	<0.0001	0.0007		0.0001	0.0001	0.0002	<0.0001
HES6 M	coefficient r	0.7839	0.6822	0.7046	0.7661	0.8048	0.6439		0.6745	0.6687	0,8829
	p-value	<0.0001	<0.0001	<0.0001	<0.0001	<0.0001	0.0001		<0.0001	<0.0001	<0.0001
VEGF-A NM	coefficient r	0.8920	0.7511	0.6287	0.8012	0.6233	0.6057	0.6745		0.7989	0,8260
	p-value	<0.0001	<0.0001	0.0001	<0.0001	0.0001	0.0001	<0.0001		<0.0001	<0.0001
VEGF-A M	coefficient r	0.8734	0.7519	0.6845	0.8117	0.6443	0.5956	0.6687	0.7989		0,7681
	p-value	<0.0001	<0.0001	<0.0001	<0.0001	<0.0001	<0.0001	<0.0001	<0.0001		<0.0001
MVD	coefficient r	0.8787	0.7169	0.7424	0.8564	0.7368	0.7020	0.8829	0.8260	0.7681	
	p-value	<0.0001	<0.0001	<0.0001	<0.0001	<0.0001	<0.0001	<0.0001	<0.0001	<0.0001	

Table S1: Correlation matrix of markers for ligand and target gene of Notch pathway and angiogenesis quantified in MM patients' BM biopsies. 34 BMPs from MM patients were analyzed at the onset of the disease prior to drug treatments. Quantified parameters were used for a Pearson correlation analysis using GraphPad Prism 6. Statistical analysis was carried out using two-tailed *t*-test. Table shows a correlation matrix with coefficient r and *p*-value.

© 2020 by the authors. Licensee MDPI, Basel, Switzerland. This article is an open access article distributed under the terms and conditions of the Creative Commons Attribution (CC BY) license (http://creativecommons.org/licenses/by/4.0/).
